# Overexpression of TIGIT in NK and T Cells Contributes to Tumor Immune Escape in Myelodysplastic Syndromes

**DOI:** 10.3389/fonc.2020.01595

**Published:** 2020-08-07

**Authors:** Fanqiao Meng, Lijuan Li, Fengzhu Lu, Jing Yue, Zhaoyun Liu, Wei Zhang, Rong Fu

**Affiliations:** Department of Hematology, Tianjin Medical University General Hospital, Tianjin, China

**Keywords:** cancer immunotherapy, CD155, CD226, MDS, TIGIT, PD-1

## Abstract

**Objective:**

Targeting immune checkpoints, such as PD-1, represents a promising approach for cancer immunotherapy, achieving long-term disease remission rates in numerous types of cancer. T cell immunoglobulin and ITIM domain (TIGIT) is a checkpoint receptor associated with the antitumor roles of NK and T cells. Notably, the blockade of TIGIT has been revealed as a potential promising approach in cancer immunotherapy. However, the therapeutic potential of blocking TIGIT in myelodysplastic syndrome (MDS) remains unclear and further research is required to reveal their role.

**Methods:**

Fresh peripheral blood (PB) and bone marrow (BM) were obtained from patients with MDS and healthy donors (HDs) at the Tianjin Medical University General Hospital between January 21 2018 and March 22 2019. The present study investigated the expression levels of TIGIT on NK and T cells using flow cytometry (FCM) and PCR. In addition, other checkpoint receptors, such as CD226 and PD-1, were also investigated. To determine the mechanisms of antitumor immunity, the functions of NK and T cells expressing TIGIT were determined.

**Results:**

TIGIT was found to be highly expressed on NK and T cells of the PB, where it was involved in disease progression and the immune escape of MDS. The high expression levels of TIGIT were associated with decreased NK and T cell function, and significantly lower secretions of activation factors, such as CD107a, IFN-γ and TNF-α. Notably, blocking TIGIT enhanced the antitumor effects of NK and T cells.

**Conclusion:**

The results of the present study suggested that targeting TIGIT alone or in combination with PD-1 may be a promising anticancer therapeutic strategy in MDS.

## Introduction

Myelodysplastic syndrome (MDS) is a group of heterogeneous diseases with abnormal quality and quantity of blood cell. It originates from hematopoietic stem cell and is characterized by peripheral blood (PB) cytopenia, bone marrow (BM) dysfunctional hematopoiesis, and an increased risk of progression to acute myeloid leukemia (AML) (1–3). No gold standard exists for the diagnosis of MDS. The prognosis is worse than that of AML, and the survival period is short. Anemia, bleeding, infection, and other symptoms lead to a significant decline in the quality of life of patients, directly resulting in death (4, 5). The natural course of disease and prognosis of patients with MDS vary widely, with a life expectancy of several months or years. Hence, treatment should be individualized (6, 7). Hematopoietic cell transplantation remains the only curative treatment of MDS, but transplant-related complications increase with age, gender, cytogenetic subgroups, number of red blood cell transfusions (8). Although some progress has been made in the treatment of MDS, effective treatment for MDS is still lacking (9, 10).

T and NK cells are essential antitumor effector cells (11). The antitumor effect is regulated by both costimulatory and coinhibitory signaling molecules, such as PD-1, cytotoxic T lymphocyte antigen-1 (CTLA-4), T cell immunoglobulin and ITIM domain (TIGIT), and CD226 (12–16). TIGIT is a novel coinhibitory receptor that is widely expressed on both T and NK cells (17). Although TIGIT and CD226 both bind to CD155, they exert opposite and competitive effects; CD226 is the costimulatory counterreceptor to TIGIT; however, its affinity with CD155 is low (18). TIGIT has been reported to inhibit CD226 signaling by binding to CD155 at a higher affinity. It also directly inhibits the killing effect of NK cells on tumor cells via the ITIM region of TIGIT, which is similar to the inhibitory effect of CTLA-4 and PD-1 on T cells, as the engagement of PD-L1 with PD-1 also suppresses the function of T cells (17). Recent efforts to restore the immune response by antagonizing the inhibitory signals used by tumors have demonstrated promise. For example, blockade of the immune checkpoints PD-1, CTLA-4, and TIGIT has reported remarkable success in AML (19), lymphoma (20), MDS (21), and multiple myeloma (22). Nevertheless, the antitumor effects of TIGIT or PD-1 inhibitors alone are limited, and further studies are urgently required.

To the best of our knowledge, the potential mechanisms underlying the simultaneous activation of antitumor T and NK cells by blocking TIGIT and PD-1 has not been fully investigated. One common hypothesis is that the simultaneous blockade of TIGIT and PD-1 may represent a novel cancer immunotherapy by enhancing both T and NK cell-mediated immune responses. The data from the present study identified a potential immunosuppressive effect of TIGIT and PD-1 in MDS, and identified the mechanism of TIGIT and PD-1 in the tumor microenvironment by improving the functions of NK and T cells.

## Materials and Methods

### Patient Samples

Fresh PB and BM were obtained from patients with MDS and healthy donors (HDs) at the Tianjin Medical University General Hospital between January 21, 2018 and March 22 2019. The patients were classified based on the 2016 World Health Organization (WHO) classifications (23), according to the age, sex, international prognostic scoring system score, and cytogenetics. The study was performed according to the Declaration of Helsinki and approved by the Ethics Committee of Tianjin Medical University General Hospital. Written informed consent for participation was obtained from each individual.

### Flow Cytometry

Single-cell suspensions were stained according to standard protocols and subsequently stained for 30 min at 4°C with the following antibodies (BD Biosciences): TIGIT-FITC, CD226-FITC, PD1-FITC, CD155-PE, CD33-APC, CD34-FITC, CD3-PerCP, CD4-APC, CD8-PE, CD16-APC, CD56-BV421, and CD56-PB450. CD56^+^ NK cells were divided into CD56^+^CD16^–^ NK (CD56^*bright*^ NK) cells and CD56^+^CD16^+^ NK (CD56^*dim*^ NK) cells using an anti-human CD16 antibody (24). After washing the suspension twice, the cells were analyzed by FCM. The fluorescence compensation between channels was adjusted to circle the target cell group, and the FCM data were subsequently analyzed using Cell Quest^TM^ Pro 4.0.2 software (BD Biosciences).

### Proliferation Assay

TIGIT^+^ NK, TIGIT^+^ CD8^+^ T, and TIGIT^+^ CD4^+^ T cells were sorted by FCM and stained with 5 μmol/L carboxyfluorescein diacetate succinimidyl ester (CFSE, BD Biosciences) for 10 min. CFSE-labeled TIGIT^+^ NK, TIGIT^+^ CD8^+^ T and TIGIT^+^ CD4^+^ T cells were stimulated with 5 μg/ml anti-CD3/CD28 for 8 h. TIGIT^+^ NK, TIGIT^+^ CD8^+^ T, and TIGIT^+^ CD4^+^ T cell proliferation was evaluated by FCM.

### Cell Isolation and Culture

Peripheral blood mononuclear cells (PBMCs) and bone marrow mononuclear cells (BMMCs) were isolated using lymphocyte separation medium (Beijing Solarbio Science & Technology, Inc., China). NK, CD4^+^ T, T, and CD8^+^ T cells were isolated from PBMCs by negative selection using the human NK, T, CD4^+^T, and CD8^+^T cell isolation Kit (Miltenyi Biotec, Bergisch Gladbach, Germany). The purity of the isolated cell detected by FCM was up to 95%. CD33^+^ and CD34^+^ cells obtained from BMMCs were isolated using anti-CD33 and anti-CD34 magnetic microspheres, and LS columns according to the manufacturers’ protocols (Miltenyi Biotec GmbH). CD33^+^ and CD34^+^ cells from BMMCs were cultured at 37°C with 5% CO_2_ in Iscove’s medium (Invitrogen, Carlsbad, CA, United States) supplemented with 20% fetal bovine serum (Gibco-Invitrogen) and 100 U/mL penicillin and streptomycin (Invitrogen). The partial sample was stored at −80°C for further analysis.

### T and NK Cell Functional Assays

T and NK cell functions were analyzed by determining the secretion of cytokines (IFN-γ, TNF-α and CD107a) by FCM. T cells were stimulated with 5 μg/ml anti-CD3/CD28, whereas NK cells were stimulated with 10 ng/ml IL-12, in RPMI-1690 medium supplemented with 10% fetal calf serum for 12 h for the cytotoxicity assays (25). T and NK cells were cultured with K562 cells at an effector to target ratio of 10:1 for 8 h before staining. The cells were incubated for 10 h with 100 ng/ml phorbol myristate acetate (Sigma-Aldrich; Merck KGaA) and 2.0 μg/ml ionomycin (Sigma-Aldrich; Merck KGaA) to stimulate the production of cytokines. Then, cells were washed twice and incubated with conjugated antibodies against the following for 30 min at 4°C: CD3, CD4, CD8, CD56, TIGIT, IFN-γ, TNF-α, and CD107a. Following the incubation, the cells were washed and analyzed by FCM. To investigate the effects of blocking TIGIT alone or in combination with PD-1, purified T and NK cells were randomized into different groups and treated with PD-1 mAb or TIGIT mAb for 72 h. The levels of cytokines were then analyzed in the same manner.

### Co-cultured With CD155 of BM

TIGIT^+^ NK, TIGIT^–^ NK, TIGIT^+^ T, and TIGIT^–^ T cells were co-cultured with CD155 of BM at a 2:1 ratio in the presence of 5 μg/ml anti-CD3/CD28 and 10 ng/ml IL-12 for 3 days. Cells were then washed and incubated with conjugated antibodies against the following for 30 min at 4°C: CD3, CD56, TIGIT, IFN-γ, TNF-α, and CD107a. T and NK cell function test according to the manufacturer’s instructions.

### Reverse Transcription-Quantitative PCR (RT-qPCR)

Total RNA was extracted from isolated CD33^+^ cells from both HDs and patients with MDS using the RNeasy Mini kit (Qiagen, Inc.). Total RNA was reverse transcribed into cDNA using the NuGEN Ovation Human RNA-Seq Multiplex system (NuGEN Technologies). The mRNA expression levels of TIGIT, PD-1 and CD226 in NK and T cells were also analyzed using RT-qPCR. The forward and reverse primer sequences used for qPCR are listed in Table 1. The relative mRNA expression levels were calculated using the 2^–Δ^ ^Δ^ ^*Cq*^ method, and gene expression values were normalized to the endogenous control GAPDH.

**TABLE 1:** The primer sequences for PCR.

Target gene	Primer sequences	
	Forward primer	Reverse primer
TIGIT	5-CGTGAACGATACAGGGGAGT-3	5-GCAATGGAATCTGGAACCTG-3
CD226	5-GGCAGAAATTTCACCTCCAA-3	5-GCAAGTAGCAGCGGTAAAGC-3
PD-1	5-ACCTGGGTGTTGGGAGGGCA-3	5-GGAGTGGATAGGCCACGGCG-3
CD155	5-TCCTGTGGACAAACCAATCA-3	5-GTTACGGGACATGCCTGAGT-3
GAPDH	5-GCACCGTCAAGGCTGAGAAC-3	5-TGGTGAAGACGCCAGTGGA-3

### Statistical Analysis

Data are presented as the mean ± standard deviation (SD). Statistical differences between individual groups were analyzed using an one-way analysis of variance (ANOVA) or a Mann–Whitney *U* test on GraphPad Prism software. The correlation between the two parameters was calculated using the Spearman’s rank correlation coefficient. *P* < 0.05 was considered to indicate a statistically significant difference.

## Results

### Patient Characteristics and Sample Data

Patients with MDS and age and sex matched HDs were recruited. No significant differences were observed in the sex ratio or age between the patients with MDS and HDs (*P* > 0.05). Among MDS patient subtypes (26), including MDS with single lineage dysplasia (MDS SLD, *n* = 5), MDS with ring sideroblasts and single lineage dysplasia (MDS RS SLD, *n* = 3), MDS with multilineage dysplasia (MDS MLD, *n* = 2), MDS del(5q) (*n* = 4), MDS unclassifiable (MDS-U, *n* = 1), MDS with excess blasts 1 (MDS EB1, *n* = 11) and MDS with excess blasts 2 (MDS EB2, *n* = 14). The clinical characteristics of the patients are presented in Table 2.

**TABLE 2:** Patient and clinical characteristics.

Demographic variable	MDS (*n* = 40)
Age	
Median age, years (range)	63 (39–83)
Sex, *n* (%)	
Female	22 (55)
Male	18 (45)
IPSS risk score, *n* (%)	
Low (Int-1 and Int-2)	12 (30)
High	28 (70)
WHO 2016 classification, *n* (%)	
MDS del5q	4 (10)
MDS-U	1 (2.5)
MDS SLD	5 (12.5)
MDS RS SLD	3 (7.5)
MDS MLD	2 (5)
MDS EB1	11 (27.5)
MDS EB2	14 (35)
Karyotype, *n* (%)	
Normal	17 (42.5)
Abnormal	23 (57.5)

### Proportion and Function of NK and T Cells Is Decreased in Patients With MDS

**FIGURE 1:**
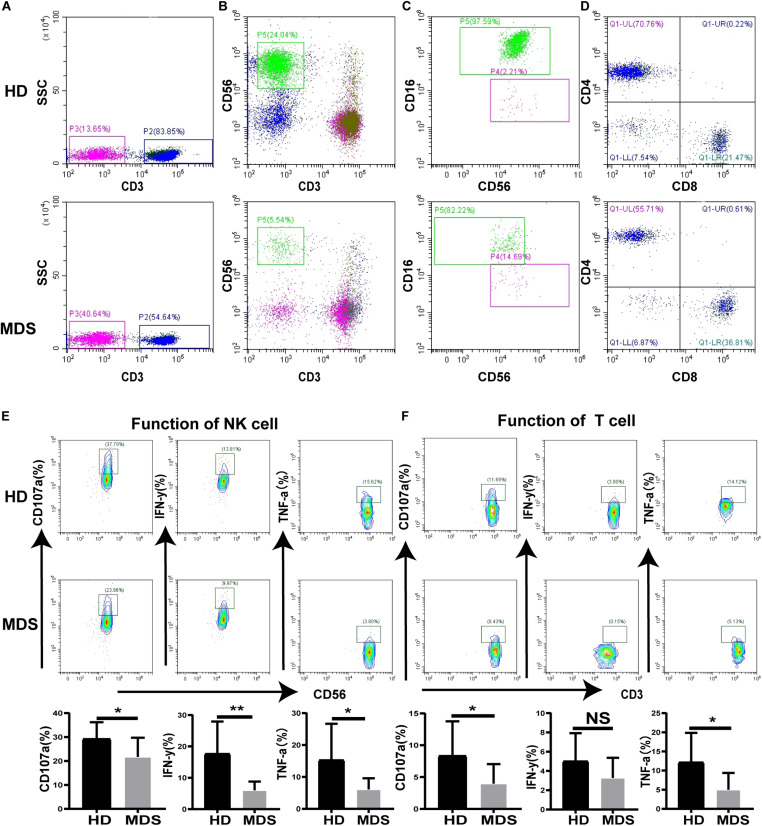
The proportion and function of T and NK cells in MDS and HDs. **(A–D)** Flow cytometry analysis of T **(A)**, NK **(B)**, NK subgroup **(C)**, T subgroup **(D)** expression levels in PB from MDS and HDs. Figure A and B shows the percentage of CD3^+^T cell and CD56^+^NK cell in all lymphocytes, respectively. Panel **(C)** shows the percentage of CD56^+^16^+^NK and CD56^+^16^–^NK cells in all CD56^+^NK cells. Panel **(D)** shows the percentage of CD3^+^CD4^+^T and CD3^+^CD8^+^T cells in all CD3^+^T cells. **(E,F)** CD107a, IFN-γ, and TNF-a were analyzed to investigate the function of NK **(E)** and T **(F)** cells between MDS and HDs. MDS, myelodysplastic syndromes; HDs, healthy donors; PB, peripheral blood; *P < 0.05, **P < 0.01, ***P < 0.005, ****P < 0.0001, NS denotes not significant; data are presented as mean ± SD, Statistical differences between HDs (*N* = 20) and MDS (*N* = 26) **(A–D)**, HDs (*N* = 10) and MDS (*N* = 10) **(E–F)** were determined by Mann–Whitney unpaired *t*-test.

To investigate the effects of NK and T cells in MDS, the frequency and absolute numbers of NK and T cells in the PB were analyzed using FCM. The absolute counts for NK and T cell were calculated by multiplying the NK and T cell percentage by the absolute count of the gated population/100. The absolute cell numbers of NK (180.8 ± 231.5 cells/μL vs. 431.9 ± 247.5 cells/μL, *p* < 0.0001) and T cells (775.9 ± 378.8 cells/μL vs. 1218 ± 623.5 cells/μL *p* = 0.0154) was lower in patients with MDS compared with the HDs (Table 3). The levels of NK and T cells were significantly decreased in patients with MDS compared with the HDs (Figures 1A,B). In addition, the NK and T cell subgroups were also examined. Notably, significantly increased percentages of CD56^*bright*^ NK and CD4^+^ T cells were identified in patients with MDS compared with the HDs, while the percentages of CD56^*dim*^ NK and CD8^+^ T cells observed were significantly decreased (Figures 1C,D). The levels of NK and T cells are presented in Table 3. Subsequently, the secretory levels of CD107a, IFN-γ and TNF-α were analyzed to determine whether there were differences in the functions of NK and T cells between the patients with MDS and HDs (Figures 1E,F). Similar results were obtained, with T and NK cells also exhibiting decreased CD107a, IFN-γ and TNF-α levels in the patients with MDS, indicating a poor killing capability. These data indicated that the proportion and function of NK and T cells in patients with MDS may be reduced, alongside the antitumor function, which is consistent with the results of a previous study (27).

**TABLE 3:** NK, T, TIGIT, CD226, and PD-1 expression from PBMC in MDS and HDs.

	MDS	HD	*P*	Significance
CD56^+^NK (%)	5.967 ± 5.264	14.31 ± 6.148	<0.0001	****
NK cells (cells/μL)	180.8 ± 231.5	431.9 ± 247.5	<0.0001	****
TIGIT^+^NK (%)	26.97 ± 15.10	10.50 ± 10.10	0.0003	***
CD226^+^NK (%)	62.21 ± 16.22	86.16 ± 7.447	<0.0001	****
CD16^+^NK (%)	72.73 ± 20.17	92.12 ± 2.835	<0.0001	****
CD16^–^NK (%)	22.12 ± 19.96	4.686 ± 2.355	<0.0001	****
CD3^+^T (%)	53.02 ± 17.89	73.93 ± 11.04	<0.0001	****
T cells (cells/μL)	775.9 ± 378.8	1218 ± 623.5	0.0154	*
TIGIT^+^T (%)	35.42 ± 15.55	18.32 ± 23.38	0.0006	***
CD226^+^T (%)	53.85 ± 13.70	70.96 ± 10.25	<0.0001	****
PD-1^+^T (%)	31.11 ± 15.62	12.73 ± 8.841	<0.0001	****
CD4^+^T (%)	56.13 ± 14.61	38.17 ± 12.09	0.0002	***
TIGIT^+^CD4^+^T (%)	18.18 ± 9.187	15.05 ± 15.43	0.1002	NS
CD226^+^CD4^+^T (%)	59.47 ± 19.65	67.03 ± 14.19	0.293	NS
PD-1^+^CD4^+^T (%)	17.53 ± 7.731	11.64 ± 13.15	0.0127	*
CD3^+^CD8^+^T (%)	35.02 ± 10.79	52.48 ± 11.10	<0.0001	****
TIGIT^+^CD8^+^T (%)	49.41 ± 19.58	13.97 ± 14.76	<0.0001	****
CD226^+^CD8^+^T (%)	60.46 ± 12.81	73.37 ± 11.79	0.0007	***
PD-1^+^CD8^+^T (%)	47.88 ± 20.22	13.19 ± 13.03	<0.0001	****

MDS, myelodysplastic syndromes; HDs, healthy donors; PBMCs, peripheral blood mononuclear cells; *P < 0.05, **P < 0.01, ***P < 0.005, ****P < 0.0001, NS denotes not significant; data are presented as mean ± SD using an unpaired *t*-test. NK and T cell expression were analyzed between HDs (*N* = 20) and MDS (*N* = 26). The expression of TIGIT, CD226, and PD-1 were analyzed between HDs (*N* = 20) and MDS (*N* = 30).

### TIGIT, CD226, and PD-1 Are Aberrantly Expressed in NK and T Cells of Patients With MDS

**FIGURE 2:**
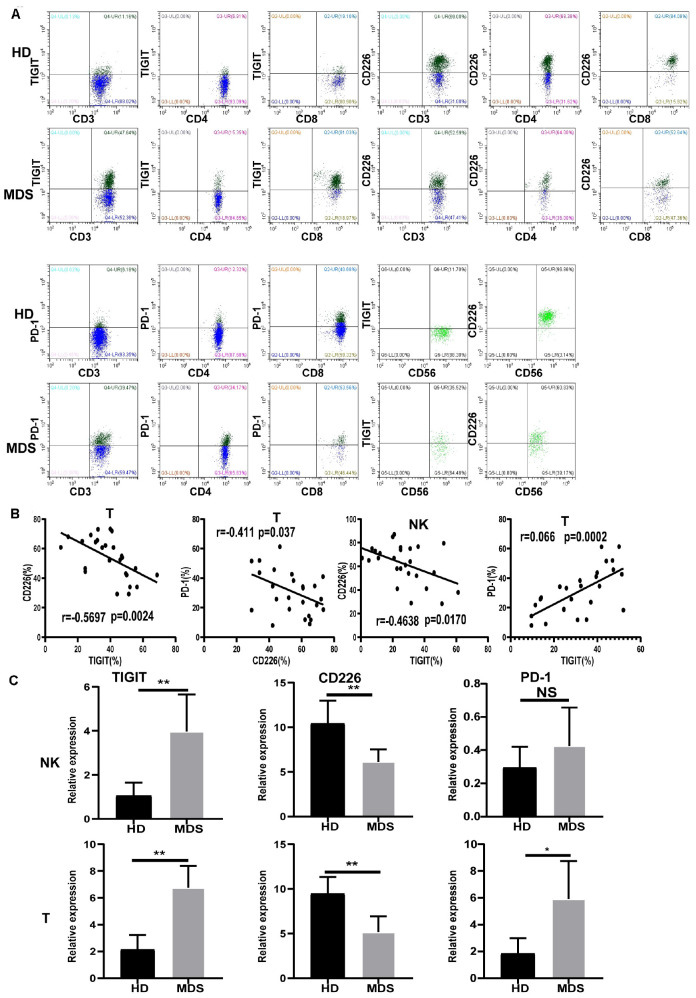
The expression of TIGIT, PD-1, and CD226 on T and NK and correlation in MDS and HD. Flow cytometry and statistical analysis of TIGIT, PD-1, and CD226 on T and NK in MDS and HDs by flow cytometry **(A)** and PCR **(C)**. Correlation between TIGIT, CD226, and PD-1 expression level in MDS **(B)**. Data are pooled from two independently acquired sets of samples. MDS, myelodysplastic syndromes; HDs, healthy donors; PB, peripheral blood; *P < 0.05, **P < 0.01, ***P < 0.005, ****P < 0.0001, NS, denotes not significant; data are presented as mean ± SD, Statistical differences between HD (*N* = 20) and MDS (*N* = 30) were determined by an unpaired *t*-test. Correlation coefficients (*r*) and *p* are indicated in the figure.

To investigate whether TIGIT may serve as a potential target in MDS, the levels of TIGIT in patients with MDS and HDs were analyzed by FCM (Figure 2A). Interestingly, the T and NK cells from patients with MDS had decreased expression levels of CD226 and increased expression levels of TIGIT compared with the HDs. A significantly increased frequency of PD-1 expression on T cells was also observed. However, there were no significant differences identified in the PD-1 expression levels on NK cells between patients with MDS and HDs (data not shown). The reason for these findings may be that PD-1 is rarely expressed on NK cells, with expression levels of < 5% reported. In addition, no significant differences were determined in the TIGIT and CD226 expression levels in CD4^+^ T cells; however, a significant difference was found in CD8^+^ T cells compared with HDs (Table 3). These results are consistent with a previous study, which demonstrated that TIGIT exerted a suppressive effect over the CD8^+^ T cell response in AML (28). Of note, TIGIT and PD-1 expression levels were found to be significantly increased on CD8^+^ T cells compared with CD4^+^ T cells in patients with MDS, whereas no significant differences were observed for CD226 expression levels between CD8^+^ T and CD4^+^ T cells (Table 3). Previously, TIGIT was reported to be highly expressed on CD8^+^ T cells in myeloma, indicating that TIGIT mainly acts on T cells by regulating CD8^+^ T cells (22).

### Correlation Between TIGIT, CD226, and PD-1 Expression Levels

The expression levels of TIGIT, CD226, and PD-1 in MDS were analyzed by FCM to investigate their associations. In patients with MDS, the expression levels of TIGIT and PD-1 were found to be negatively correlated with the expression levels of CD226, whereas the expression levels of TIGIT were positively correlated with PD-1 (Figure 2B). In addition, significant differences were discovered in TIGIT, CD226 and PD-1 expression levels between patients with MDS and the HDs. Moreover, further analysis also identified significant differences in the patients with MDS (Figure 2B and Table 3). These results revealed that high TIGIT expression levels were associated with higher PD-1 and lower CD226 expression levels. The reason for these findings may be that TIGIT and PD-1 are inhibitory receptors, while CD226 is an activatory receptor. A previous study reported that the increased percentage of PD-1 on T cells may be associated with lower antileukemia effects and a poor prognosis for AML (29). As TIGIT and PD-1 are mediators of T and NK cell depletion, these findings suggested that the elevated TIGIT and PD-1 expression levels on the NK and T cells may indicate a suppressive environment in MDS, promoting immune evasion.

### mRNA Expression Levels of TIGIT, CD226, and PD-1

The mRNA expression levels of TIGIT, CD226, and PD-1 were analyzed using RT-qPCR in NK and T cells. Patients with MDS exhibited upregulated expression levels of TIGIT and PD-1, and downregulated expression levels of CD226, compared with the HDs (Figure 2C). In conclusion, these results demonstrated that the expression levels of TIGIT, PD-1, and CD226 on NK and T cells were strongly associated with the immune response in MDS.

### Expression of TIGIT, PD-1, and CD226 on NK and T Cells Is Associated With the Disease Status

TIGIT and PD-1 were found to be overexpressed in several types of solid tumor, which was subsequently associated with a poor prognosis (30, 31). Thus, the expression of PD-1, TIGIT, and CD226 derived from lower-risk and higher-risk patients with MDS was analyzed. Relatively increased TIGIT and PD-1 expression levels, and decreased CD226 expression levels were found in high-risk patients with MDS (Table 4). Next, whether the decreased proportion and absolute numbers of NK and T cells in MDS was associated with the disease status, a significantly higher percentage and absolute numbers of NK and T cells were identified in the lower-risk patients with MDS (Table 4). All of these results are presented in Table 4. These results indicated that TIGIT, PD-1, and CD226 may be associated with the disease status and may be used as an approach to predict the prognosis of MDS.

**TABLE 4:** TIGIT, CD226, and PD-1 expression from PBMC in MDS.

	Low Risk (*N* = 11)	High Risk (*N* = 15)	*P*	Significance
CD56^+^NK (%)	8.639 ± 6.443	3.982 ± 2.225	0.003	**
NK cells (cells/μL)	170.3 ± 99.24	142.7 ± 299.9	0.0043	**
TIGIT^+^NK (%)	18.77 ± 10.17	34.00 ± 15.35	0.0061	**
CD226^+^NK (%)	72.70 ± 10.28	55.65 ± 15.99	0.0057	**
CD3^+^T (%)	64.78 ± 8.526	44.40 ± 18.20	0.0013	**
T cells (cells/μL)	933.7 ± 383.9	660.2 ± 383.9	0.0170	*
TIGIT^+^T (%)	20.86 ± 9.387	39.49 ± 10.46	0.0001	***
CD226^+^T (%)	61.93 ± 11.73	49.31 ± 13.39	0.0221	*
PD-1^+^T (%)	19.56 ± 10.16	39.59 ± 13.42	0.0006	***
CD33^+^CD155 (%)	47.62 ± 26.32	67.65 ± 23.74	0.1480	NS
CD34^+^CD155 (%)	42.29 ± 27.15	77.94 ± 16.21	0.0433	*

MDS, myelodysplastic syndromes; PBMCs, peripheral blood mononuclear cell; *P < 0.05, **P < 0.01, ***P < 0.005; data are presented as mean ± SD using an unpaired *t*-test. The expression of TIGIT, CD226, and PD-1 were analyzed in MDS between low risk (*N* = 11) and high risk (*N* = 15).

### High Expression of TIGIT Limits the Function of NK and T Cells

**FIGURE 3:**
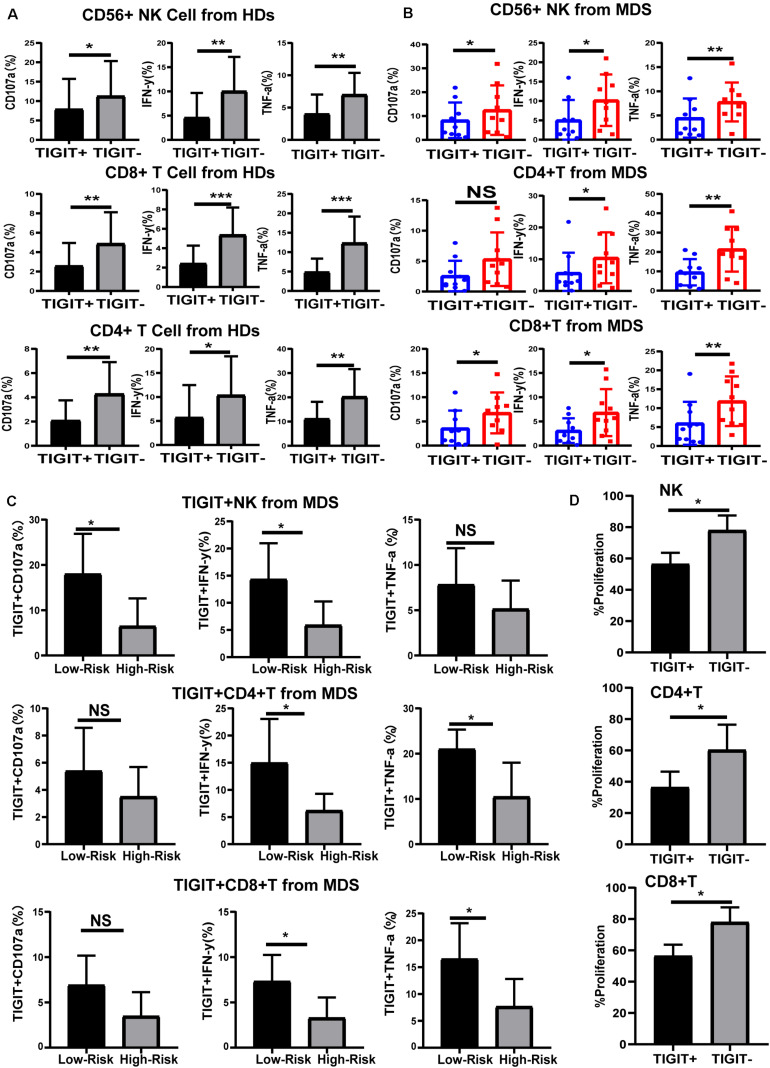
TIGIT limits the function of NK and T cells. Cytokine expression by TIGIT^+^ and TIGIT^–^ NK and T cell in HDs **(A)**, MDS **(B)**, and different risk levels MDS **(C)**. CD107a, TNF-a, and IFN-y of TIGIT^+^ and TIGIT^–^ cell was determined by flow cytometry. Statistical differences in MDS (*N* = 10) patients were determined by a paired *t*-test. TIGIT + NK, TIGIT + CD8 + T and TIGIT + CD4 + T cells proliferation **(D)**. *P < 0.05, **P < 0.01, ***P < 0.005, ****P < 0.0001.

To understand the effects of TIGIT expression on NK and T cell function in MDS, the levels of cytokines were investigated. TIGIT^+^ NK, TIGIT^+^ CD8^+^ T, and TIGIT^+^ CD4^+^ T cells exhibited decreased CD107a, IFN-γ, and TNF-α expression levels compared with TIGIT-cells in HDs and MDS (Figures 3A,B), suggesting a functional impairment of TIGIT^+^ T and TIGIT^+^ NK cells. In addition, TIGIT^+^ cells from lower-risk and higher-risk patients with MDS were evaluated for the expression of CD107a, IFN-γ and TNF-α on NK and T cells. TIGIT^+^ NK and T cell from higher-risk patients exhibited decreased CD107a, IFN-γ, and TNF-α expression levels compared with lower-risk patients (Figure 3C). To define the effects of TIGIT on NK and T cell proliferation, TIGIT^+^ NK, TIGIT^+^ CD8^+^ T, and TIGIT^+^ CD4^+^ T cells proliferation was evaluated by FCM (Figure 3D). We found that the proliferation of TIGIT^+^ T and NK cells was significantly increased compared to TIGIT^–^T and NK cells. These results indicated that TIGIT may act as a negative immune checkpoint in MDS, inhibit the secretion and proliferation of cytokines and inhibit the antitumor immune response of MDS.

### CD155 Is Overexpressed in CD33^+^ and CD34^+^ Cells of the BM in MDS

**FIGURE 4:**
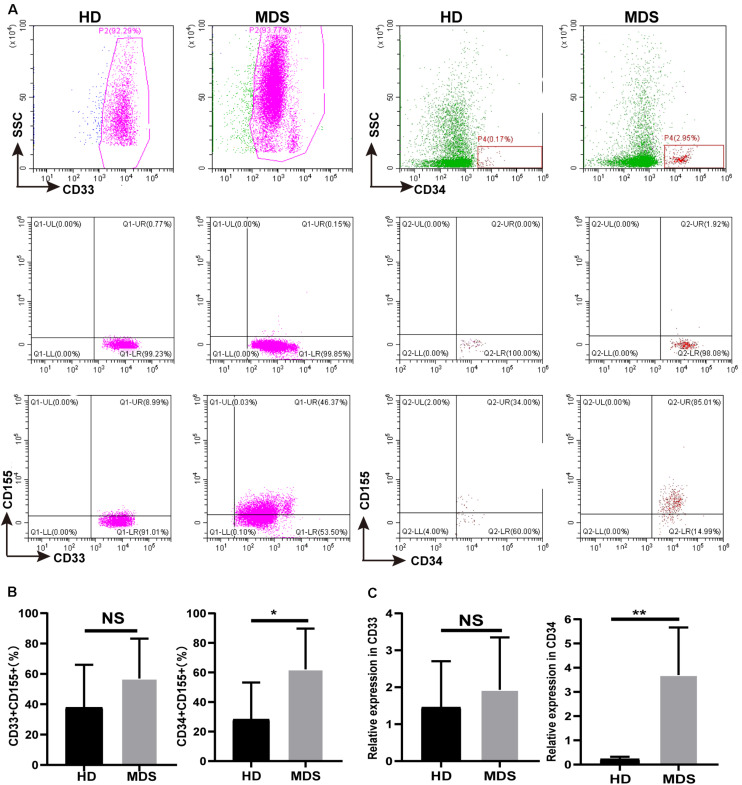
**(A)** CD155 is overexpressed in CD33^+^ and CD34^+^ cell in BM of MDS patients. The CD155 expression in CD33^+^ and CD34^+^ in BM by flow cytometry between HDs and MDS. **(B)** Statistical analysis of CD155 expression in CD33^+^ and CD34^+^ in BM between HDs and MDS. **(C)** qRT-PCR analysis of CD155 expression in CD33^+^ and CD34^+^ from MDS patients. MDS, myelodysplastic syndromes; HDs, healthy donors; BM, bone marrow; *P < 0.05, NS denotes not significant; data are presented as mean ± SD, Statistical differences were determined by Mann–Whitney unpaired *t*-test.

TIGIT has been shown to bind to CD155, a coinhibitory ligand overexpressed on multiple types of malignant tumor, where it was discovered to promote tumor progression. CD155 expression on tumor-infiltrating myeloid cells was revealed to have a negative effect on the immune antitumor response, which is conducive to immune evasion (32). Thus, CD155 expression levels on CD33^+^ and CD34^+^ cells of the BM in patients with MDS were analyzed using FCM (Figure 4A). An increased trend in the percentage of CD155 was found on CD33^+^ cells, where is a significant increase in the expression levels were identified on CD34^+^ cells (Figure 4B). In addition, there was no significant differences in the expression of CD155 in CD33^+^ cells between patients with high risk and low risk, while a significant difference in CD34^+^ cells (Table 4). The mRNA expression levels of CD155 were subsequently determined using RT-qPCR in CD33^+^ and CD34^+^ cells. However, no significant differences were observed in the mRNA expression levels of CD155 in CD33^+^ cells between patients with MDS and HDs, whilst a significant difference was observed in CD34^+^ cells (Figure 4C). These data demonstrated that the CD155 expression levels in CD34^+^ cells may play a more important role in MDS, and that the CD155 expression in CD34^+^ cells may combine with TIGIT to produce an effect.

### Blocking TIGIT Alone or in Combination With PD-1 Reverses NK and T Cell Hypofunction

**FIGURE 5:**
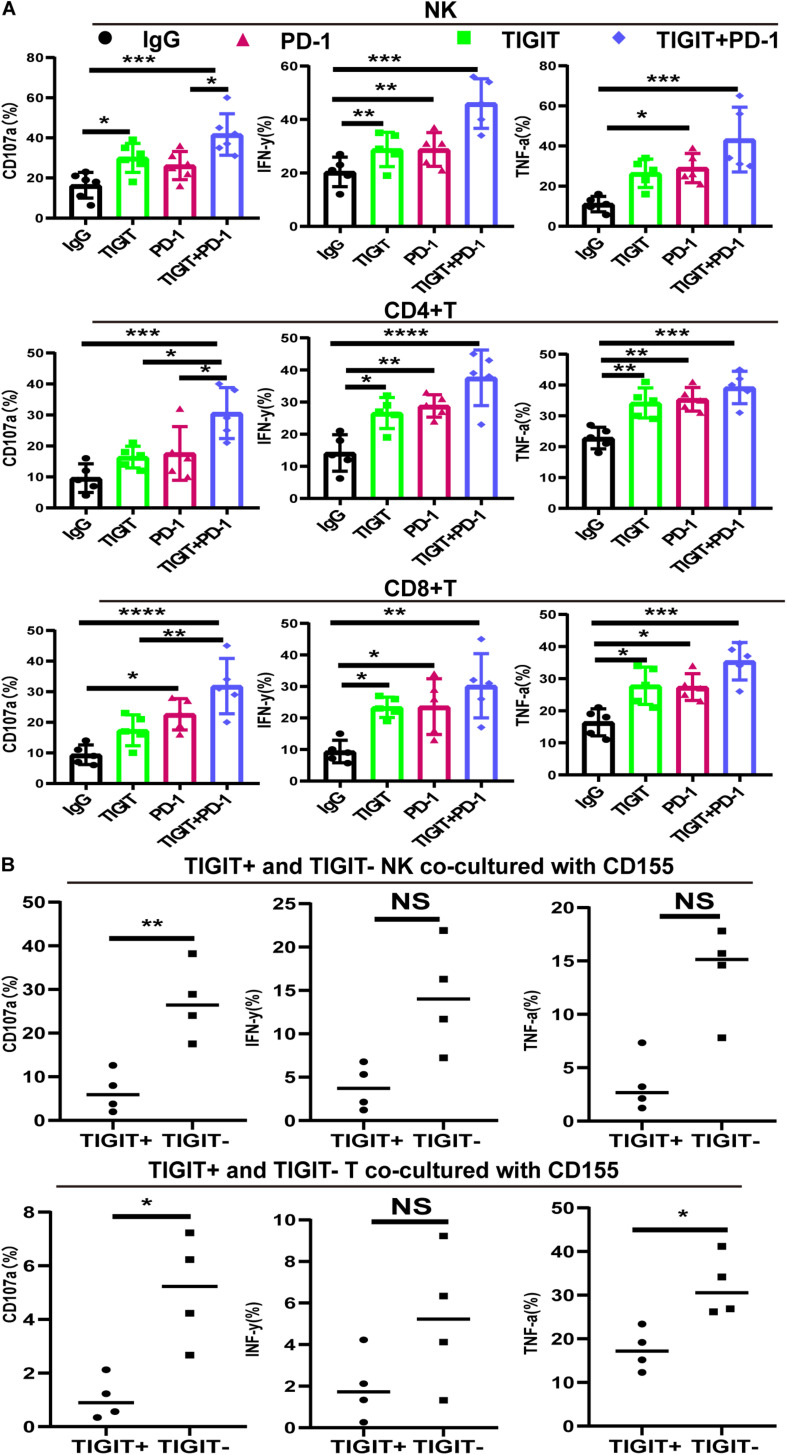
PD-1 and TIGIT combination blockade increases the function of NK and T cell in MDS patients. **(A)** TIGIT and PD-1 blockade increases the secretion of CD107a, TNF-a, and IFN-y in NK, CD8^+^T, and CD4^+^T from MDS. **(B)** TIGIT^+^ and TIGIT^–^ T from NK and T cells co-cultured with CD155 of BM. Data are presented as mean ± SD. Statistical significance was determined by non-parametric one-way ANOVA followed by Dunn’s multiple comparison test. *P < 0.05, **P < 0.01, ***P < 0.005, ****P < 0.0001.

To investigate the effects of blocking TIGIT alone or in combination with PD-1, the expression levels of CD107a, IFN-γ and TNF-α were investigated following the use of an anti-TIGIT and anti-PD-1 antibody. The expression levels of CD107a, IFN-γ and TNF-α on NK, CD8^+^ T and CD4^+^ T cells were significantly increased in response to TIGIT or PD-1 blockade, with the most significant increases observed in the combination treatment group (Figure 5A). Upregulated TIGIT expression levels have been found to be associated with NK and T cell functional exhaustion, whilst the blockade TIGIT was discovered to restore their function. These data suggested that TIGIT and PD-1 may have a key role in inhibiting cytokine release, thus suppressing T and NK cell function.

### CD155 Suppress TIGIT+ NK and TIGIT+ T Cell Function

To investigate the interaction between CD155 and TIGIT, purified CD155 of BM were co-cultured with TIGIT^+^ and TIGIT^–^ NK and T cells. The release of three different cytokines (IFN-γ, CD107a, and TNF-α) was measured by FCM. As expected, TIGIT^–^ cells co-cultured with CD155 of BM exhibited high expression of IFN-γ, CD107a and TNF-α compared to TIGIT^+^ (Figure 5B). We observed a trend towards higher IFN-γ in TIGIT^–^ cell compared to TIGIT^+^ cell but no statistically significant difference.

## Discussion

NK and T cells are antitumor effector cells found in the MDS tumor microenvironment; however, their mechanisms of promoting antitumor immunity have not been fully investigated. Immunosuppression and immune escape are key to the success of tumor immunotherapy. Immune checkpoint receptors, such as TIGIT, PD-1 and CTLA-4, have been discovered to be highly expressed in numerous types of cancer, and they are currently being targeted to improve antitumor and T or NK cell responses (33, 34). The blockade of PD-L1, TIGIT, PD-1 and CTLA-4 to enhance the effector T or NK cell-mediated antitumor immune response has demonstrated early clinical promise as immunosuppressive therapy (35, 36). However, an urgent problem remains in the fact that the side effects of the drugs are significant and the curative rate remains low. Furthermore, to the best of our knowledge, evidence supporting the functional role of TIGIT in MDS is currently lacking.

**FIGURE 6:**
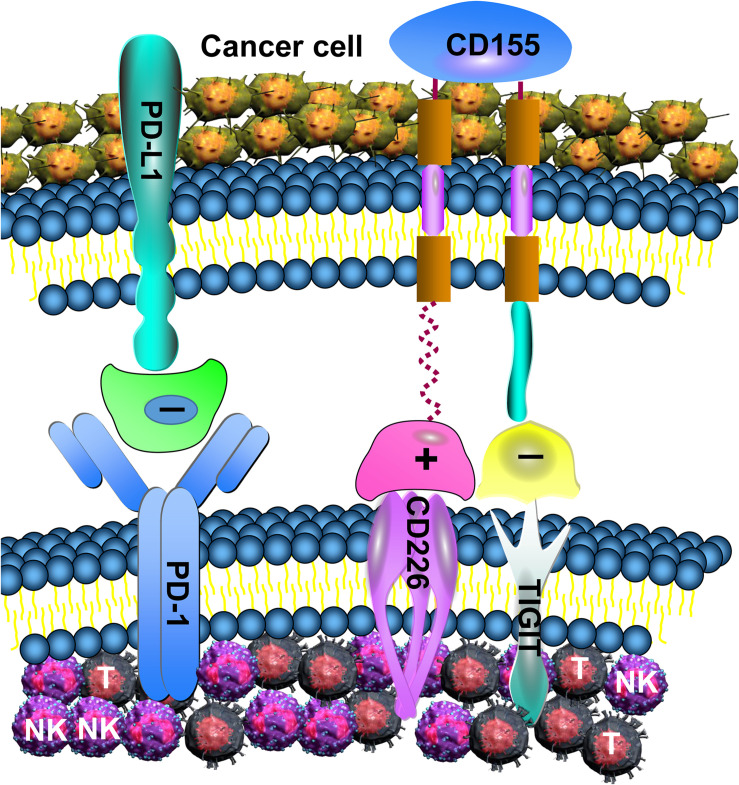
TIGIT, PD-1, and CD226 in T and NK cell mediate the immune response. Increased expression of TIGIT and PD-1 in NK and T cell induces excessive negative regulation, leading to decreased anti-tumor function. Although CD226 can enhance the anti-tumor effect of NK and T cell, its binding power with CD155 is low, and TIGIT can inhibit the binding of CD226 with CD155.

In the present study, TIGIT, CD226, CD155, and PD-1 were all discovered to be aberrantly expressed in MDS. The downregulation of CD226 and the overexpression of TIGIT and PD-1 on NK and T cells contributed to reducing CD107a, IFN-γ and TNF-α secretion, in addition to being associated with immunosuppression. It is worthy to note that the IFN-γ and TNF-α levels detected in the present study were secreted by NK and T cells of PB, rather than in the microenvironment. By contrast, in a previous study, IFN-γ and TNF-α levels were discovered to be elevated in the BM of patients with MDS, and inhibition of the microenvironment led to ineffective hematopoiesis (37). In addition, other cells in the BM, such as mesenchymal stem cells and monocytes, also secrete these cytokines, which can also cause them to rise. An imbalance between TIGIT and CD226 may lead to the progression of MDS. The current study discovered that TIGIT, CD226, and PD-1 were all involved in the MDS-mediated tumor immune response (Figure 6). Expression of TIGIT, PD-1,CD155 and CD226 is associated with the disease status, and more highly expressed in high-risk MDS.

Importantly, blocking TIGIT significantly increased function of NK and T cell, the combined treatment with PD-1 further activated NK and T cells and produced a greater antitumor response. These results were consistent with a previous study, which demonstrated that the blockade of TIGIT and PD-L1 markedly enhanced T and NK function, and resulted in stronger antitumor effects (38). The dual inhibition of TIGIT and PD-1 has been further investigated in an attempt to elicit potent antitumor CD8^+^ T cell responses in patients with advanced melanoma (39). In addition, compared with the NK cells, blocking TIGIT alone or in combination with PD-1 caused T cells to produce more cytokines, indicating that this inhibitory effect mainly acts on T cells. The reason for this may be that although TIGIT and PD-1 are co-expressed on T and NK cells, they are found at higher levels on T cells, especially PD-1. These results suggested that activation of TIGIT and PD-1 may reduce NK and T cell cytotoxicity and cytokine production. Consistently, blockade of PD-1 and TIGIT results in sustained immunity in tumor models (12). This novel finding provides a strong theoretical basis for the combined blockade of TIGIT and PD-1 to enhance the antitumor response in patients with MDS. Our results also show that TIGIT suppress NK and T cell proliferation and cytokines production. Notably, the present findings may provide evidence to suggest targeting TIGIT may be beneficial for the treatment of MDS.

The present study also indicated that the expression of NK cells and expression of TIGIT on NK cells may be critical to the clinical outcomes of TIGIT based immunotherapy. However, because TIGIT acts on both NK and T cells, it was not possible to determine which cell type plays a greater role in the antitumor immunity in the present study, thus further investigations are required. Altogether, the present research provided evidence for the role of TIGIT in the pathogenesis of MDS, alongside suggesting a strong theoretical basis for the development of novel therapies targeting TIGIT to restore T and NK cell functions. However, direct evidence supporting the clinical role of TIGIT in patients with MDS was not provided due to the lack of animal validation. Thus, future studies should aim to test the effectiveness and tolerance of TIGIT in *in vivo* models before the results can be applied in the clinic.

## Conclusion

In conclusion, the findings of the present study demonstrated that the overexpression of TIGIT inhibited the activity of NK and T cells in patients with MDS, contributing to tumor immune escape. Notably, blocking TIGIT was demonstrated to reverse the depletion of NK and T cells. Furthermore, the dual blockade of TIGIT and PD-1 was observed to be more effective in improving T and NK cell functions and antitumor immunity. The decrease in the percentage and function of NK and T cells in patients with MDS was associated with abnormal expression of TIGIT, CD155, and PD-1, and low expression of CD226. In summary, targeting TIGIT alone or combination with PD-1 may be a promising anticancer treatment strategy in MDS.

## Data Availability Statement

The datasets generated for this study are available on request to the corresponding author.

## Ethics Statement

The study was performed according to the Declaration of Helsinki and approved by the Ethics Committee of Tianjin Medical University General Hospital. Written informed consent to participate was obtained from each individual.

## Author Contributions

FM and WZ designed the research study, performed the analysis, and wrote the manuscript. JY, ZL, and FL abstracted the data and assisted in the collection and analysis of the data. LL, RF, and WZ critically revised the manuscript, performed the data analysis, and ensured correct analysis of the data. All authors contributed to the article and approved the submitted version.

## Conflict of Interest

The authors declare that the research was conducted in the absence of any commercial or financial relationships that could be construed as a potential conflict of interest.

## References

[1] Garcia-Manero G Fenaux P. Hypomethylating agents and other novel strategies in myelodysplastic syndromes. *J Clin Oncol.* (2011) 29:516–23. 10.1200/JCO.2010.31.0854 21220589 PMC3056493

[2] Garcia-Manero G. Myelodysplastic syndromes: 2015 Update on diagnosis, risk-stratification and management. *Am J Hematol.* (2015) 90:831–41. 10.1002/ajh.24102 26294090

[3] Jabbour E Takahashi K Wang X Cornelison AM Abruzzo L Kadia T Acquisition of cytogenetic abnormalities in patients with IPSS defined lower-risk myelodysplastic syndrome is associated with poor prognosis and transformation to acute myelogenous leukemia. *Am J Hematol.* (2013) 88:831–7. 10.1002/ajh.23513 23760779 PMC3923606

[4] Kim SY Park Y Kim H Kim J Kwon GC Koo SH. Discriminating myelodysplastic syndrome and other myeloid malignancies from non-clonal disorders by multiparametric analysis of automated cell data. *Clin Chim Acta.* (2018) 480:56–64. 10.1016/j.cca.2018.01.029 29378171

[5] Kattamis A Aydinok Y Taher A. Optimising management of deferasirox therapy for patients with transfusion-dependent thalassaemia and lower-risk myelodysplastic syndromes. *Eur J Haematol.* (2018) 101:272–82. 10.1111/ejh.13111 29904950

[6] Zeidan AM Faltas B Douglas Smith B Gore S. Myelodysplastic syndromes: what do hospitalists need to know? *J Hosp Med.* (2013) 8:351–7. 10.1002/jhm.2049 23666619 PMC4234094

[7] Sekeres MA. Epidemiology, natural history, and practice patterns of patients with myelodysplastic syndromes in 2010. *J Natl Compr Canc Netw.* (2011) 9:57–63. 10.6004/jnccn.2011.0006 21233244

[8] Ishiyama K Aoki J Itonaga H Uchida N Takahashi S Ohno Y Graft-versus-MDS effect after unrelated cord blood transplantation: a retrospective analysis of 752 patients registered at the Japanese Data Center for Hematopoietic Cell Transplantation. *Blood Cancer J.* (2019) 9:31. 10.1038/s41408-019-0192-x 30842405 PMC6403210

[9] Patel B Hirsch C Clemente M Sekeres M Makishima H Maciejewski JP. Genetic and molecular characterization of myelodysplastic syndromes and related myeloid neoplasms. *Int J Hematol.* (2015) 101:213–8. 10.1007/s12185-015-1747-7 25690487

[10] Stahl M Zeidan AM. Hypomethylating agents in combination with histone deacetylase inhibitors in higher risk myelodysplastic syndromes: Is there a light at the end of the tunnel? *Cancer.* (2017) 123:911–4. 10.1002/cncr.30532 28094843

[11] Krijgsman D de Vries NL Skovbo A Andersen MN Swets M Bastiaannet E Characterization of circulating T-, NK-, and NKT cell subsets in patients with colorectal cancer: the peripheral blood immune cell profile. *Cancer Immunol Immunother.* (2019) 68:1011–24. 10.1007/s00262-019-02343-7 31053876 PMC6529387

[12] Zhang Q Bi J Zheng X Chen Y Wang H Wu W Blockade of the checkpoint receptor TIGIT prevents NK cell exhaustion and elicits potent anti-tumor immunity. *Nat Immunol.* (2018) 19:723–32. 10.1038/s41590-018-0132-0 29915296

[13] Hung AL Maxwell R Theodros D Belcaid Z Mathios D Luksik AS TIGIT and PD-1 dual checkpoint blockade enhances antitumor immunity and survival in GBM. *Oncoimmunology.* (2018) 7:e1466769. 10.1080/2162402X.2018.1466769 30221069 PMC6136875

[14] Lozano E Dominguez-Villar M Kuchroo V Hafler DA. The TIGIT/CD226 axis regulates human T cell function. *J Immunol.* (2012) 188:3869–75. 10.4049/jimmunol.1103627 22427644 PMC3324669

[15] Puri S Shafique M. Combination checkpoint inhibitors for treatment of nonsmall-cell lung cancer: an update on dual anti-CTLA-4 and anti-PD-1/PD-L1 therapies. *Drugs Context.* 2020 9:2019-9-2. 10.7573/dic.2019-9-2 32158484 PMC7048109

[16] Agdashian D ElGindi M Xie C Sandhu M Pratt D Kleiner DE The effect of anti-CTLA4 treatment on peripheral and intra-tumoral T cells in patients with hepatocellular carcinoma. *Cancer Immunol Immunother.* (2019) 68:599–608. 10.1007/s00262-019-02299-8 30688989 PMC6662600

[17] Manieri NA Chiang EY Grogan JL. TIGIT: a key inhibitor of the cancer immunity cycle. *Trends Immunol.* (2017) 38:20–8. 10.1016/j.it.2016.10.002 27793572

[18] Sanchez-Correa B Valhondo I Hassouneh F Lopez-Sejas N Pera A Bergua JM DNAM-1 and the TIGIT/PVRIG/TACTILE Axis: novel immune checkpoints for natural killer cell-based cancer immunotherapy. *Cancers.* (2019) 11:877. 10.3390/cancers11060877 31234588 PMC6628015

[19] Zhu L Kong Y Zhang J Claxton DF Ehmann WC Rybka WB Blimp-1 impairs T cell function via upregulation of TIGIT and PD-1 in patients with acute myeloid leukemia. *J Hematol Oncol.* (2017) 10:124. 10.1186/s13045-017-0486-z 28629373 PMC5477125

[20] Armand P Chen YB Redd RA Joyce RM Bsat J Jeter E PD-1 blockade with pembrolizumab for classical Hodgkin lymphoma after autologous stem cell transplantation. *Blood.* (2019) 134:22–9. 10.1182/blood.2019000215 30952672 PMC6609955

[21] Sarhan D Brandt L Felices M Guldevall K Lenvik T Hinderlie P 161533 TriKE stimulates NK-cell function to overcome myeloid-derived suppressor cells in MDS. *Blood Adv.* (2018) 2:1459–69. 10.1182/bloodadvances.2017012369 29941459 PMC6020813

[22] Guillerey C Harjunpaa H Carrie N Kassem S Teo T Miles K TIGIT immune checkpoint blockade restores CD8(+) T-cell immunity against multiple myeloma. *Blood.* (2018) 132:1689–94. 10.1182/blood-2018-01-825265 29986909

[23] Arber DA Orazi A Hasserjian R Thiele J Borowitz MJ Le Beau MM The 2016 revision to the World Health Organization classification of myeloid neoplasms and acute leukemia. *Blood.* (2016) 127:2391–405. 10.1182/blood-2016-03-643544 27069254

[24] Poli A Michel T Theresine M Andres E Hentges F Zimmer J. CD56bright natural killer (NK) cells: an important NK cell subset. *Immunology.* (2009) 126:458–65. 10.1111/j.1365-2567.2008.03027.x 19278419 PMC2673358

[25] Zhao XY Yu XX Xu ZL Cao XH Huo MR Zhao XS Donor and host coexpressing KIR ligands promote NK education after allogeneic hematopoietic stem cell transplantation. *Blood Adv.* (2019) 3:4312–25. 10.1182/bloodadvances.2019000242 31869417 PMC6929384

[26] Greenberg PL Stone RM Al-Kali A Barta SK Bejar R Bennett JM Myelodysplastic syndromes, version 2.2017, NCCN clinical practice guidelines in oncology. *J Natl Compr Canc Netw.* (2017) 15:60–87. 10.6004/jnccn.2017.0007 28040720

[27] Hejazi M Manser AR Frobel J Kundgen A Zhao X Schonberg K Impaired cytotoxicity associated with defective natural killer cell differentiation in myelodysplastic syndromes. *Haematologica.* (2015) 100:643–52. 10.3324/haematol.2014.118679 25682594 PMC4420213

[28] Kong Y Zhu L Schell TD Zhang J Claxton DF Ehmann WC T-Cell Immunoglobulin and ITIM domain (TIGIT) associates with CD8+ T-cell exhaustion and poor clinical outcome in AML patients. *Clin Cancer Res.* (2016) 22:3057–66. 10.1158/1078-0432.CCR-15-2626 26763253

[29] Huang J Tan J Chen Y Huang S Xu L Zhang Y A skewed distribution and increased PD-1+Vbeta+CD4+/CD8+ T cells in patients with acute myeloid leukemia. *J Leukoc Biol.* (2019) 106:725–32. 10.1002/JLB.MA0119-021R 31136687

[30] Sun Y Luo J Chen Y Cui J Lei Y Cui Y Combined evaluation of the expression status of CD155 and TIGIT plays an important role in the prognosis of LUAD (lung adenocarcinoma). *Int Immunopharmacol.* (2020) 80:106198. 10.1016/j.intimp.2020.106198 31954274

[31] Four M Cacheux V Tempier A Platero D Fabbro M Marin G PD1 and PDL1 expression in primary central nervous system diffuse large B-cell lymphoma are frequent and expression of PD1 predicts poor survival. *Hematol Oncol.* (2017) 35:487–96. 10.1002/hon.2375 27966264

[32] Kucan Brlic P Lenac Rovis T Cinamon G Tsukerman P Mandelboim O Jonjic S. Targeting PVR (CD155) and its receptors in anti-tumor therapy. *Cell Mol Immunol.* (2019) 16:40–52. 10.1038/s41423-018-0168-y 30275538 PMC6318332

[33] Harjunpaa H Guillerey C. TIGIT as an emerging immune checkpoint. *Clin Exp Immunol.* (2019) 200:108–19. 10.1111/cei.13407 31828774 PMC7160651

[34] Wang B Zhang W Jankovic V Golubov J Poon P Oswald EM Combination cancer immunotherapy targeting PD-1 and GITR can rescue CD8(+) T cell dysfunction and maintain memory phenotype. *Sci Immunol.* (2018) 3:eaat7061. 10.1126/sciimmunol.aat7061 30389797

[35] Yang H Bueso-Ramos C DiNardo C Estecio MR Davanlou M Geng QR Expression of PD-L1, PD-L2, PD-1 and CTLA4 in myelodysplastic syndromes is enhanced by treatment with hypomethylating agents. *Leukemia.* (2014) 28:1280–8. 10.1038/leu.2013.355 24270737 PMC4032802

[36] Wu L Mao L Liu JF Chen L Yu GT Yang LL Blockade of TIGIT/CD155 signaling reverses T-cell exhaustion and enhances antitumor capability in head and neck squamous cell carcinoma. *Cancer Immunol Res.* (2019) 7:1700–13. 10.1158/2326-6066.CIR-18-0725 31387897

[37] Xiong H Yang XY Han J Wang Q Zou ZL. Cytokine expression patterns and mesenchymal stem cell karyotypes from the bone marrow microenvironment of patients with myelodysplastic syndromes. *Braz J Med Biol Res.* (2015) 48:207–13. 10.1590/1414-431X20144051 25608238 PMC4381940

[38] Johnston RJ Comps-Agrar L Hackney J Yu X Huseni M Yang Y The immunoreceptor TIGIT regulates antitumor and antiviral CD8(+) T cell effector function. *Cancer Cell.* (2014) 26:923–37. 10.1016/j.ccell.2014.10.018 25465800

[39] Chauvin JM Pagliano O Fourcade J Sun Z Wang H Sander C TIGIT and PD-1 impair tumor antigen-specific CD8(+) T cells in melanoma patients. *J Clin Invest.* (2015) 125:2046–58. 10.1172/JCI80445 25866972 PMC4463210

